# Postpartum Idiopathic Pancreatitis Complicated by Acute Necrotizing Pancreatitis

**DOI:** 10.7759/cureus.34002

**Published:** 2023-01-20

**Authors:** Reese Hofstrand, Mayank Singhal, Jagroop Doad, Ryan Watts

**Affiliations:** 1 Internal Medicine, Cape Fear Valley Medical Center, Fayetteville, USA; 2 Internal Medicine, Campbell University School of Osteopathic Medicine, Lillington, USA; 3 Cardiothoracic Surgery, Campbell University School of Osteopathic Medicine, Lillington, USA

**Keywords:** post partum, mrcp, ct, microlithiasis, pancreatitis

## Abstract

Acute pancreatitis (AP) is a common medical condition with a wide variety of etiologies. One of the common but frequently undetected causes of acute pancreatitis is microlithiasis, which can appear as biliary “sludge” in the gallbladder on imaging. While a broad workup should be initiated, endoscopic retrograde cholangiopancreatography (ERCP) is the gold standard for the diagnosis of microlithiasis. In this case, we present a severe presentation of acute pancreatitis in a teenager within the postpartum period. A 19-year-old woman presented with severe 10 out of 10 right upper quadrant (RUQ) pain with episodes of nausea that radiated to her back. She had no history of chronic alcoholism, illicit drug use, or over-the-counter supplement use, and no familial history of autoimmune disease, or pancreatitis. The patient was diagnosed with necrotizing acute pancreatitis with gallbladder "sludge" using contrast-enhanced computed tomography (CT) and magnetic resonance cholangiopancreatography (MRCP). She followed up with gastroenterology and had a great clinical recovery. Therefore, it is important to consider acute pancreatitis in patients with idiopathic pancreatitis in their postpartum period as they are prone to forming gallbladder "sludge" which can precipitate and cause a variation in gallbladder pancreatitis which can be difficult to detect on imaging.

## Introduction

Acute ‘idiopathic’ pancreatitis has silently become one of the leading causes of pancreatitis in the United States, especially rising within the pediatric population and elderly populations. Acute pancreatitis (AP) has an annual incidence across the USA of five to 80 persons per 100,000 of the general population. However, the incidence of AP in pregnancy remains low and is approximately one in 1,000 to one in 10,000 births. Of these select few cases, 30% are known to present in the postpartum period [1]. The most common causes of AP include gallstones and alcohol abuse, but many patients often present with microlithiasis, also known as biliary sludge [2]. Microlithiasis likely represents an early stage of cholesterol gallstone disease and approximately 20% of patients with microlithiasis eventually develop cholesterol gallstones [3]. In pregnancy and postpartum periods, the presentation of AP often ranges from mild pancreatitis to severe complications like necrosis. In this case, we report an occurrence of AP in the postpartum period presenting with microlithiasis that, to our knowledge, has not been reported and discuss its management.

## Case presentation

A previously healthy 19-year-old teen, three months postpartum, presented to the emergency room with epigastric abdominal pain, nausea, and vomiting without jaundice. Her past medical history was notable for an emergency room visit two days prior to admission. In the interim, the patient’s nausea and vomiting worsened. 

**Figure 1:**
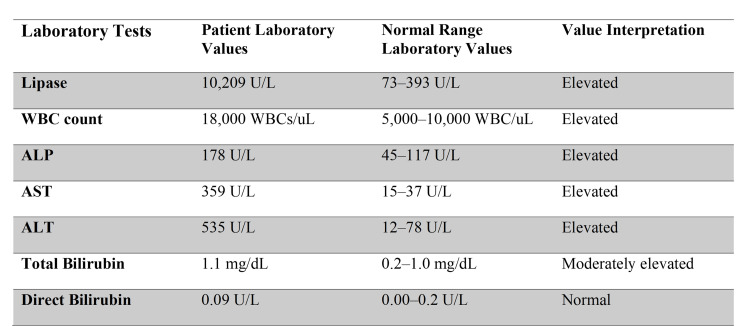
Patient laboratory values on presentation

**Figure 2:**
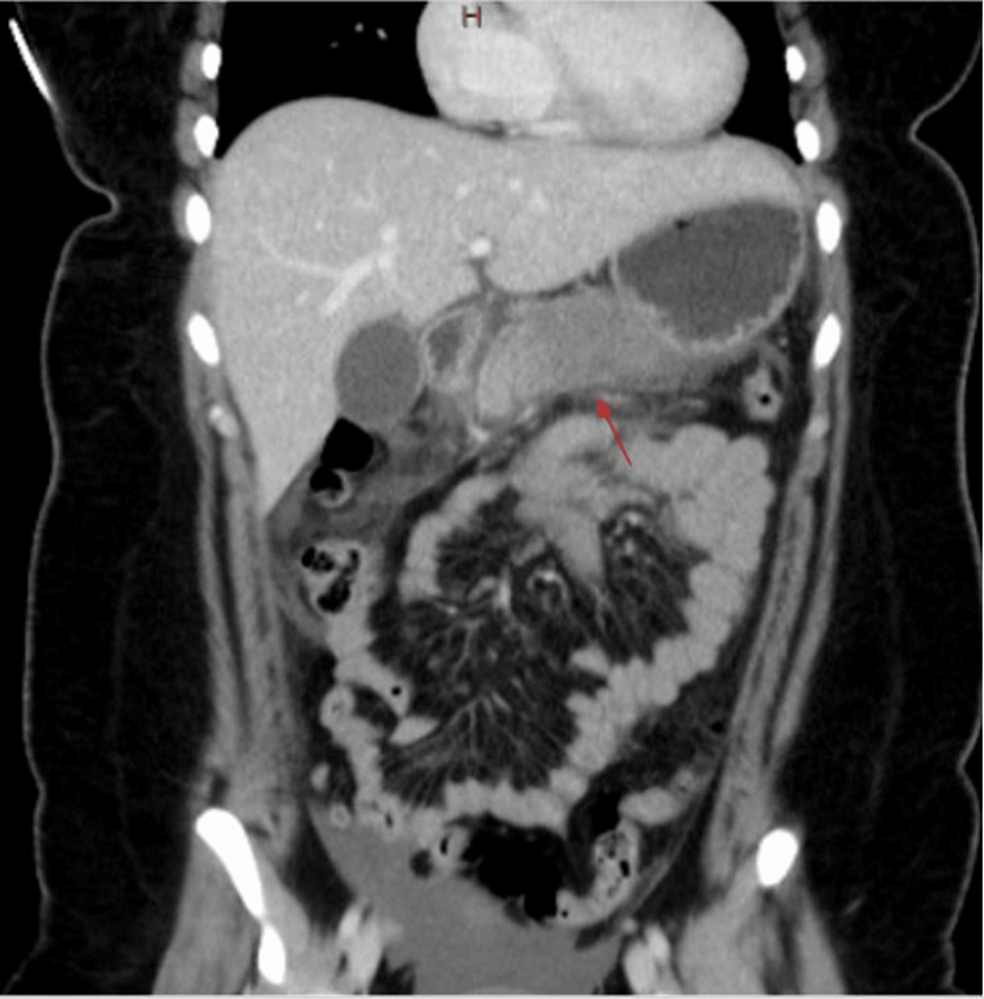
CT of the abdomen and pelvis with contrast The red arrow shows pancreatic edema

On arrival at the emergency department, the patient was initially afebrile, with a heart rate of 68 beats/minute, blood pressure of 142/85 mmHg, respiratory rate of 23 breaths/minute, and oxygen saturation of 99% on room air. Preliminary investigation revealed an elevated lipase level of 10,209 U/L (normal range: 73-393 U/L) with a white blood cell count of 18,000 WBCs/μl (normal range: 5,000 to 10,000 WBCs/μl). Her clinical presentation was very characteristic of a patient with severe pancreatitis. Additionally, the patient had elevated liver function tests with alkaline phosphatase (ALP) of 178 U/L (normal range: 45-117 U/L), aspartate transferase (AST) of 359 U/L (normal range: 15-37 U/L), alanine transaminase (ALT) of 535 U/L (normal range: 12-78 U/L), international normalized ratio (INR) 1.2 (normal range: 0.9-1.1), PT 12.6 seconds (normal range: 9.7-12.4 seconds), activated partial thromboplastin time (APTT) 31.1 seconds (normal range: 24.3-34.6 seconds), cortisol 18.4 ug/dL (normal range: 6.2-19.4 ug/dL), thyroid-stimulating hormone (TSH) 0.677 uIU/mL (normal range: 0.463-3.980 uIU/mL), total bilirubin of 1.1 mg/dL (0.2-1.0 mg/dL), and a direct bilirubin of 0.09 U/L (normal range: 0.00-0.20 U/L) (Figure 1). The lipid panel drawn at the presentation showed triglycerides 56 mg/dL (normal range <150 mg/dL) and immunoglobulin (Ig)G subclass 4 25 (normal range 3-104 mg/dL). Initial ultrasound (US) gallbladder was nondiagnostic due to an abundance of bowel gas. Computed tomography (CT) of the abdomen and pelvis with contrast (Figure 2) performed on hospital day one showed no cholelithiasis, biliary tree dilatation, or gallbladder wall thickening.

**Figure 3:**
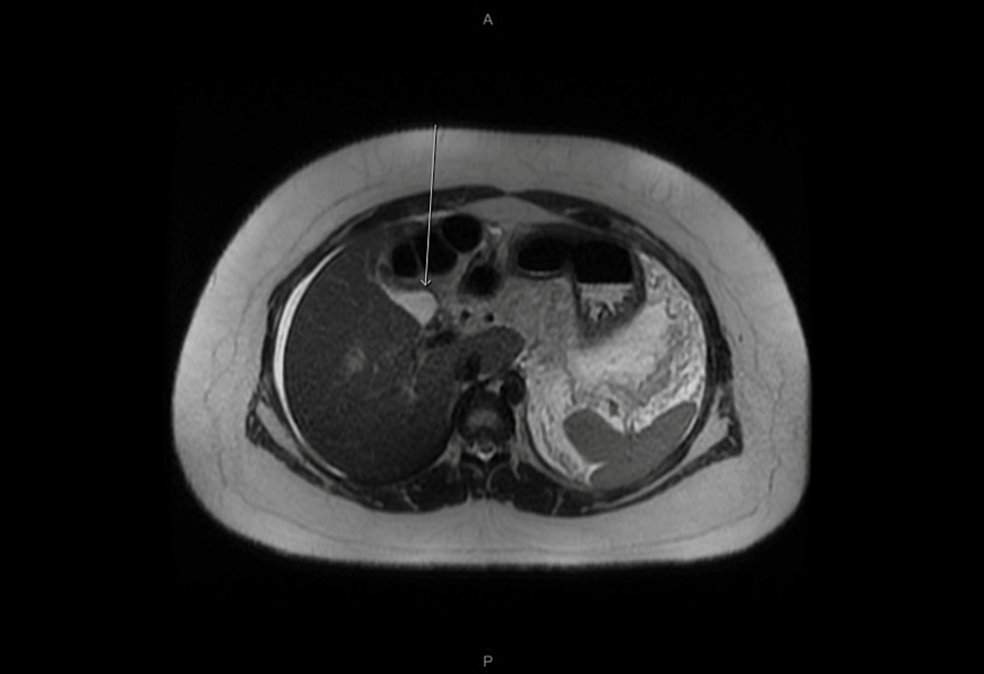
Heavily T2 weighed MRCP (image 1) MRCP: Magnetic resonance cholangiopancreatography The white arrow shows the normal gallbladder

**Figure 4:**
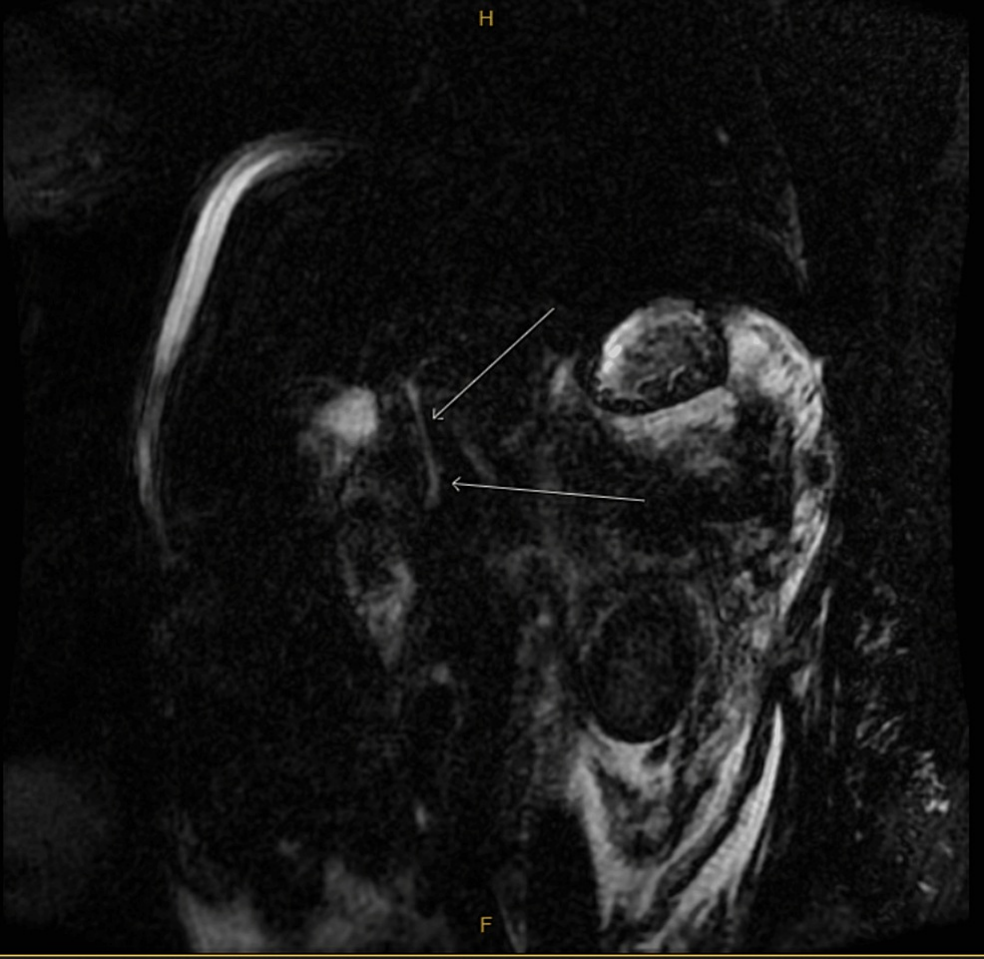
Heavily T2 weighted MRCP (image 2) MRCP: Magnetic resonance cholangiopancreatography The white arrows show the normal common bile duct

**Figure 5:**
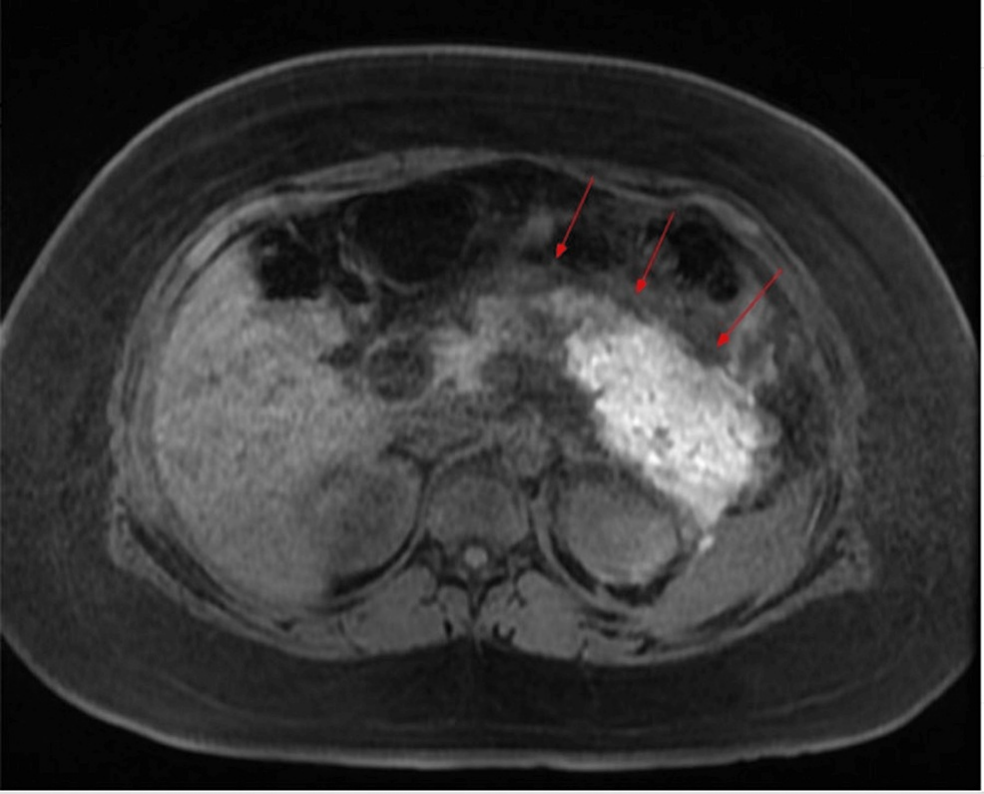
Heavily T2 weighted MRCP (image 3) MRCP: Magnetic resonance cholangiopancreatography The image shows stigmata of pancreatic necrosis, pancreatic hemorrhage, and retroperitoneal edema (red arrows pointing to edema and hyperintensity representing hemorrhage)

**Figure 6:**
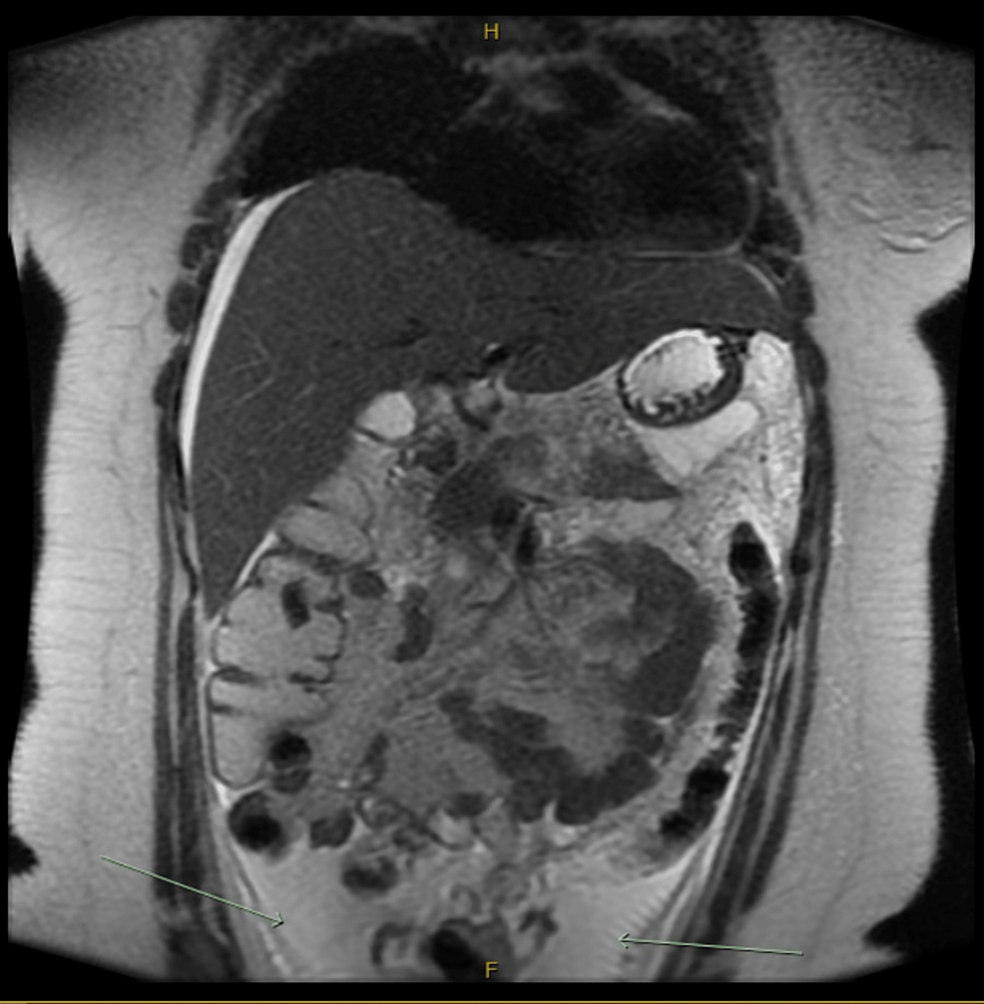
Heavily T2 weighted MRCP (image 4) MRCP: Magnetic resonance cholangiopancreatography The arrows show ascites

The following day the patient developed tachycardia and a concerning new onset fever of 102.7°F. An infectious workup was drawn including blood culture, chest X-ray, and urine culture were not consistent with infection. She was started on meropenem empirically for possible ascending cholangitis. Gastroenterology was subsequently consulted and they recommended ERCP, which required transfer to another advanced endoscopic center. Prior to the transfer, magnetic resonance cholangiopancreatography (MRCP) was performed at our center on hospital day two which displayed necrosis of the pancreatic body and tail with increasing retroperitoneal edema. Figures 3-6 depict MRCP findings. 

She was eventually accepted to a tertiary care center for further management. After the transfer to the tertiary care center, antibiotics were continued and emergent ERCP was postponed as the patient showed signs of clinical improvement. She was managed with pain medications. Lipase downtrended from 10,209 to 5,346, AST/ALT/alkaline phosphatase downtrended to the normal range (peak values are 630, 695, and 214 U/L respectively), and her fever improved with antibiotics over the next several days. Initial and follow-up blood cultures were negative for any infection. Meropenem was held and the patient was monitored clinically without antibiotics. ERCP was eventually canceled as she continued to improve clinically and it was presumed that her biliary pathology had spontaneously resolved. 

Per gastrointestinal (GI) experts, the most likely cause of pancreatitis given significant liver function tests was biliary in origin. Given the lack of stones on imaging, proximity to childbirth, lack of autoimmune, substance use, or family history of pancreatitis, the most likely etiology of pancreatitis was microlithiasis. General surgery saw the patient and decided against doing elective cholecystectomy as no stones were identified on MRCP. She was discharged on hospital day six with outpatient GI follow-up.

## Discussion

**Figure 7:**
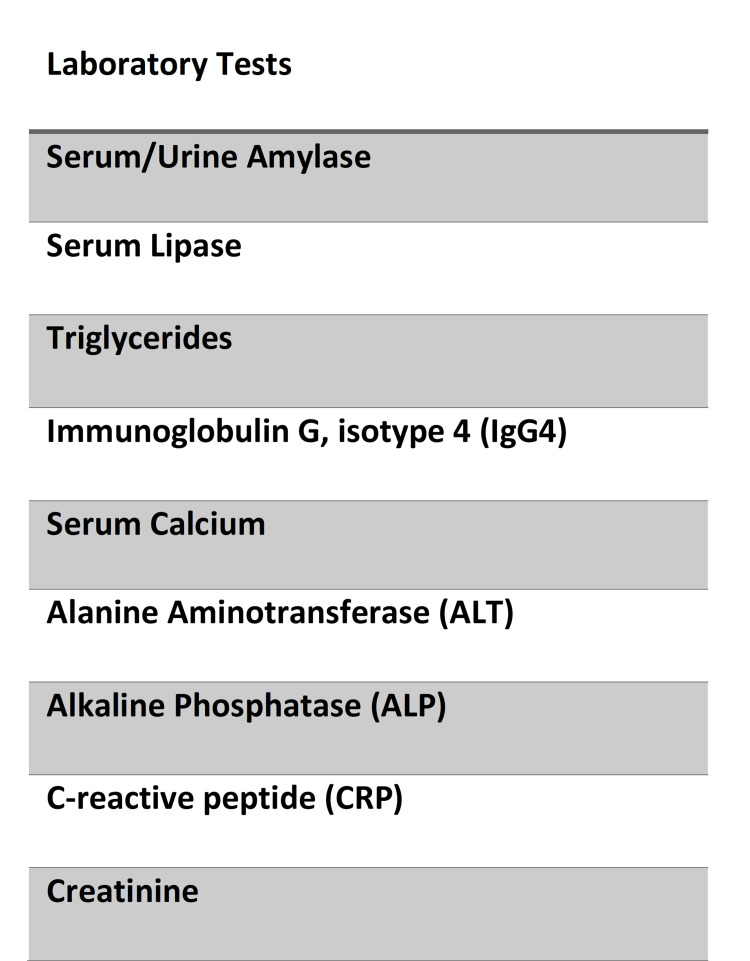
Laboratory workup for acute pancreatitis

Acute pancreatitis is inflammation of the pancreas that can present itself as acute or chronic. Typical findings often include fever, nausea, vomiting, elevated pancreatic enzymes, and abdominal pain that can radiate to the back. The most common etiologies in the Western World are alcohol consumption, gallstones, and idiopathic pancreatitis but less common etiologies should be tested for as well (Figure 7). 

‘Idiopathic’ acute pancreatitis (IP) is considered the deadliest with a mortality rate almost double that of gallstone pancreatitis, as there is often no direct etiology that can help medical professionals to direct therapy and further aid prognosis [4].

Microlithiasis is often the underlying cause of IP in a significant number of patients. The presence of symptoms frequently seen in cholelithiasis may be absent in microlithiasis. Microlithiasis refers to the presence of stones of < 3 mm in diameter, which are difficult to detect on traditional imaging. It is often described with the term ‘biliary sludge’ and may or may not be associated with other gallstones [3]. The major differentiation in pathology between gallstones and microlithiasis is that microlithiasis results from the rapid precipitation of crystals from cholesterol-supersaturated bile, which can cause symptoms when they form creating biliary dysfunction. Fracchia et al. analyzed bile composition in several patients, showing that the mechanism of these crystals may be due to a deficiency in phosphatidylcholine [5]. Moreover, Rosmorduc et al. showed that this deficiency is the direct cause of the rapid cholesterol crystallization seen in the hepatic bile of these patients. This may also be attributed to a deficiency in a multidrug resistance protein known as the *MDR3* gene, which encodes for a phosphatidylcholine translocator protein at the canalicular membrane of the hepatocyte [6]. A subsequent study supported this claim, showing that acute IP caused by microlithiasis was associated with point mutations in *MDR3*. Data specifically on postpartum and microlithiasis-related recurrence are unavailable. The terms "microlithiasis", "postpartum", "acute idiopathic pancreatitis recurrence", and "biliary sludge" were utilized. In retrospect, testing this patient for this gene may have shed additional light on the etiology given the lack of other risk factors. Microlithiasis can present with a more fulminant course, with at least half of patients experiencing complications before diagnosis, such as pancreatitis, gangrene, perforation, and peritonitis. Therefore, in patients with acute or chronic pancreatitis without clear etiology, it should raise clinical suspicion for microlithiasis [7].

Microlithiasis can be definitively diagnosed using bile microscopy, which often shows the presence of cholesterol monohydrate, calcium bilirubinate, and/or calcium carbonate crystals [4,8]. Bile can be obtained while cannulating the bile duct during endoscopic retrograde cholangiopancreatography (ERCP) or following cholecystokinin (CCK) upon stimulation during an esophagogastroduodenoscopy [9,10]. Ko et al. noted two or more crystals per 100x field, or more than four crystals per sample should be noted as a positive result [9]. In this case, having this procedure done would have clenched the diagnosis if it were available at the time of presentation.

The gold standard for imaging cholesterol microlithiasis in patients is ERCP [8]. However, transabdominal ultrasound is still the first choice of imaging because of its wide availability and low cost [11]. Cross-sectional imaging is often not necessary but may be of use in patients with necrosis [12]. Endoscopic ultrasound (EUS) is only available at properly equipped hospitals, but EUS provides a unique opportunity to swiftly diagnose pancreatitis by directly evaluating both the ducts and further characterize pancreatic anatomy. Furthermore, EUS can then lead to an easy transition into ERCP within the same procedure if necessary for sphincterotomy or removal of stones that may have been missed on imaging. In this case, the capabilities were not present on admission and the transfer facility deferred the procedure due to clinical improvement.

ERCP with bile aspiration was shown to have a sensitivity of 83% in the detection of microlithiasis, where gallbladder bile is preferred over ductal bile [8,13]. This is because transit throughout the hepatic and common bile ducts can often be too hasty to allow for the rapid formation of these crystals, thus making them extremely difficult to analyze. When performing this procedure, it is also necessary to collect bile samples prior to the administration of the contrast agent in order to avoid the formation of ‘pseudomicrolithiasis’ from contrast precipitates.

Treatment for microlithiasis is based on clinical presentation and management of symptoms and complications. While there is not much data on recurrence rates for microlithiasis-related recurrence specifically, 12.5% of these patients can develop cholelithiasis in the future [14] In patients with microlithiasis-induced pancreatitis, cholecystitis, or cholangitis, treatment includes cholecystectomy or biliary sphincterotomy depending on clinical indication. Cholecystectomy specifically has been shown to prevent episodes of pancreatitis, partially due to hepatic bile having 4-5 times less concentration of crystals than gall bladder fluid [2,8,11]. Particularly for recurrent outflow obstruction causing recurrent cholangitis or pancreatitis, it is possible to treat with endoscopic papillotomy. This is a procedure in which the sphincter muscles of the ampulla of Vater are cauterized by a radiofrequency current. This allows the enlargement of the common duct opening into the duodenum, permitting greater ease of spontaneous or surgical passage of common bile duct stones, microliths, and sludge [15]. Maintenance therapy with bile acid dissolution therapy such as ursodeoxycholic acid may be used as well [2].

## Conclusions

We presented a case of necrotizing AP with microlithiasis, which developed in a patient’s post-partum period at our center. Microlithiasis of pancreatic origin is an underdiagnosed etiology that can lead to a range of complications. Early diagnosis and ERCP can help to identify and treat this patient population appropriately. As further research is completed on this condition, preventative measures may be an option in the future to reduce episodes of idiopathic pancreatitis, especially in pregnant and postpartum females.
